# Tiam1 Transgenic Mice Display Increased Tumor Invasive and Metastatic Potential of Colorectal Cancer after 1,2-Dimethylhydrazine Treatment

**DOI:** 10.1371/journal.pone.0073077

**Published:** 2013-09-12

**Authors:** Li-Na Yu, Qing-Ling Zhang, Xin Li, Xing Hua, Yan-Mei Cui, Nian-Jie Zhang, Wen-Ting Liao, Yan-Qing Ding

**Affiliations:** 1 Department of Pathology, Nanfang Hospital, Southern Medical University, Guangzhou, Guangdong, China; 2 Department of Pathology, School of Basic Medical Sciences, Southern Medical University, Guangzhou, Guangdong, China; 3 Guangdong Provincial Key Laboratory of Molecular Tumor Pathology, Guangzhou, Guangdong, China; 4 Department of Pathology, the Forth Affiliated Hospital of Jinan University, Guangzhou, Guangdong, China; 5 Department of Pathology, Guangzhou Red Cross Hospital, Guangzhou, Guangdong, China; The University of Hong Kong, China

## Abstract

**Background:**

T lymphoma invasion and metastasis 1 (Tiam1) is a potential modifier of tumor development and progression. Our previous study in vitro and in nude mice suggested a promotion role of Tiam1 on invasion and metastasis of colorectal cancer (CRC). In the present study, we generated Tiam1/C1199-CopGFP transgenic mice to investigate the tumorigenetic, invasive and metastatic alterations in the colon and rectum of wild-type and Tiam1 transgenic mice under 1,2-dimethylhydrazine (DMH) treatment.

**Methods:**

Transgenic mice were produced by the method of pronuclear microinlectlon. Whole-body fluorescence imaging (Lighttools, Edmonton, Alberta, Canada), PCR, and immunohistochemical techniques (IHC) were applied sequentially to identify the transgenic mice. The carcinogen DMH (20 mg/kg) was used to induce colorectal tumors though intraperitoneal (i.p.) injections once a week for 24 weeks from the age of 4 weeks on Tiam1 transgenic or non-transgenic mice.

**Results:**

We successfully generated Tiam1/C1199-CopGFP transgenic mice and induced primary tumors in the intestine of both wild type and Tiam1 transgenic mice by DMH treatment. In addition, Tiam1 transgenic mice developed larger and more aggressive neoplasm than wild-type mice. Moreover, immunohistochemical staining revealed that upregulation of Tiam1 was correlated with increased expression of β-Catenin and Vimentin, and downregulation of E-Cadherin in these mice.

**Conclusions:**

Our study has provided in vivo evidence supporting that Tiam1 promotes invasion and metastasis of CRC, most probably through activation of Wnt/β-catenin signaling pathway, in a Tiam1 transgenic mouse model.

## Introduction

Colorectal cancer (CRC) contributes the third most cancer type in the western world. Recurrence and metastasis are the leading causes of death of CRC. About 50 percent of patients with CRC will die because of complication of metastasis. Although researches have identified multiple genes that are responsible for the development of CRC, molecular events that are involved in the complex processes of CRC metastasis remain largely unknown.

T lymphoma invasion and metastasis 1 (Tiam1), a guanine nucleotide exchange factor that selectively activates the Rho-like GTPase Rac, was originally identified as an invasion-inducing and metastasis-inducing gene in T lymphoma cells [1]. Tiam1 is a potential modifier of tumor initiation and progression. It was proved to be involved in Rac-regulation of actin polymerization, cell adhesion and motility, cell survival and cell cycle progression [2], [3]. However, investigations have suggested that Tiam1 has opposite effects on different cancers. Some studies demonstrated that upregulation of Tiam1 is correlated to aggressive behavior of human cancer and poor clinical outcome of patients in several types of malignant tumors, such as breast cancer, colon cancer, prostate cancer, liver cancer, nasopharyngeal carcinoma, as well as esophageal squamous cell carcinoma (ESCC) [4], [5], [6], [7], [8], [9]. While, conversely, Tiam1 potentiates homotypic cell-cell adhesion and inhibits invasion in renal cell carcinoma cells [10]. In addition, Tiam1 plays an invasion-suppressor role in Madin-Darby canine kidney (MDCK) cells [11]. A Tiam1 knock-out mouse is relatively resistant to chemical induction of skin tumors [12]. In tissue-engineered human skin, Tiam1 depletion in dermal fibroblasts led to enhanced invasiveness of epidermal keratinocytes with premalignant features [13].

Our previous study demonstrated that Tiam1 was upregulated in CRC tissues and high expression level of Tiam1 was closely associated with aggressive and metastatic potential in CRC. Silencing of Tiam1 in CRC cell lines resulted in inhibition of cell migration and invasion in vitro, and metastasis in nude mice [9]. In order to better understand the role and mechanism of Tiam1 in progression of CRC, we generated pCDFl-Tiaml-copGFP transgenic mice and investigated the influence of Tiaml on the development of carcinomas in the colon and rectum that were induced by the chemical carcinogen (DMH), in combined with histological observation of local invasion and distant metastasis.

## Materials and Methods

### Ethics statement

This study was carried out in strict accordance with the recommendations in the Guide for the Care and Use of Laboratory Animals of the National Institutes of Health. The protocol was approved by the Committee on the Ethics of Animal Experiments of Southern Medical University (Permit Number: scxK2007 – 0005). All surgery was performed under sodium pentobarbital anesthesia, and all efforts were made to minimize suffering.

### Generation of Tiam1/C1199-CopGFP transgenic mice

The full-length cDNA of Tiam1 from C1199 was cloned into the lentivirus vector pCDF1-CopGFP. Transgenic mice were produced by the method of pronuclear microinlectlon. A total of 60 super-ovulated and 33 pseudo-pregnant ICR mice were employed in the experiment. Lentivirus containing TIAM1/EGFP gene was microinjected into pronucleus of each embryo. Embryos were cultured for 72 h in FHM medium and morula-stage embryos were transferred to the oviducts of 0–5 day post-coitus pseudopregnant. Mice were pregnant and the pups were delivered at 20–21 days.

### Identification of Tiam1/EGFP transgenic mice

Normal ICR mice were used as control. Whole-body fluorescence imaging (Lighttools, Edmonton, Alberta, Canada), PCR, and immunohistochemical techniques (IHC) were applied sequentially to identify the transgenic mice. Transgenic mice and their non-transgenic littermate animals were genotyped by PCR using the following primers for Tiam1: 5′ AAGACGTACTCAGGCCATGTCC 3′ and 5′ GACCCAAATGTCGCAGTCAG 3′. Genomic DNA was prepared from mouse-tail biopsies. The PCR temperature profile was 94°C for 45 s, 58°C for 45 s, and 72°C for 45 s with extension of the last cycle for 10 min at 72°C. PCR products were analyzed by agarose gel electrophoresis with ethidium bromide under UV light. The PCR-analysis revealed that 60 of the 124 animals were transgenic. IHC was performed as previously described [9]. Rabbit polyclonal antibody against Tiam1 (Santa Cruz, CA, USA, dilution 1:200), mouse anti-β-Catenin (BD, MO, 1:500), mouse anti-E-Cadherin (BD, MO, 1:500), mouse anti-Vimentin (Cell Signaling Technology, PO, 1:200) were used.

### RNA extraction and reverse transcription-PCR (RT-PCR)

Total RNA was extracted using Trizol reagent (Invitrogen) according to the manufacturer's instructions. The RNAwas pretreated with DNase and used for cDNAsynthesis with random hexamers. The mRNA of Tiam1 was PCR amplified from cDNA samples of transgenic mice and non-transgenic animals. The following primers were used for amplification of Tiam1: sense primer, 5-AAGACGrACTCAGGCCATGTCC-3; antisense primer, 5-GACCCAAATGTCGCAGTCA-3. Glyceraldehyde-3-phosphate dehydrogenase was amplified as an internal control using sense primer, 5-AATCCCATCACCATCTTCCA-3, and antisense primer, 5-CCTGCTTCACCACCTTCTTG-3. The appropriate size of PCR products was confirmed by agarose gel electrophoresis.

### Treatment with DMH

To investigate the impact of Tiam1 on colorectal cancer development and progression, Tiam1 transgenic and nontransgenic mice were randomized into two groups, respectively, treated either with DMH (test groups) or not (control groups). Control groups 1 and 2 were given 0.1 ml PBS i.p. once weekly for 10 weeks. Test groups were given the carcinogen DMH (20 mg/kg) through intraperitoneal (i.p.) injections once a week for 24 weeks from the age of 4 weeks on Tiam1 transgenic or non-transgenic mice. All mice had free access to tap water and a standard diet. All of the animals were observed daily for clinical signs of ill health.

## Results

### Generation and identification of Tiam1/EGFP transgenic mice

A total of 823 eggs were obtained from 60 super-ovulated mice. The Tiam1-EGFP recombined lentivirus was microinjected into the perivitelline space of 628 eggs, which were then transferred into 33 ICR pseudo-pregnant mice. 21 of the 33 pseudopregnant mice were impregnated. 67 potential Tiam1/EGFP transgenic mice were born. Five positive Tiam1-EGFP transgenic mice, identified as F0-17(♂), F0-36 (♀), F0-48 (♂), F0-52 (♀) and F0-61(♀), were generated by the pro-nuclear microinjection technique. A total of 44 positive F1 (Figure S1) and 146 positive F2 Tiam1 transgenic mice were generated by F0-36 (♀) and F0-48 (♂). All of the positive Tiam1 gene PCR sequences were matched to the Tiam1 gene sequence which is available from NCBI (data not shown).

**Figure 1:**
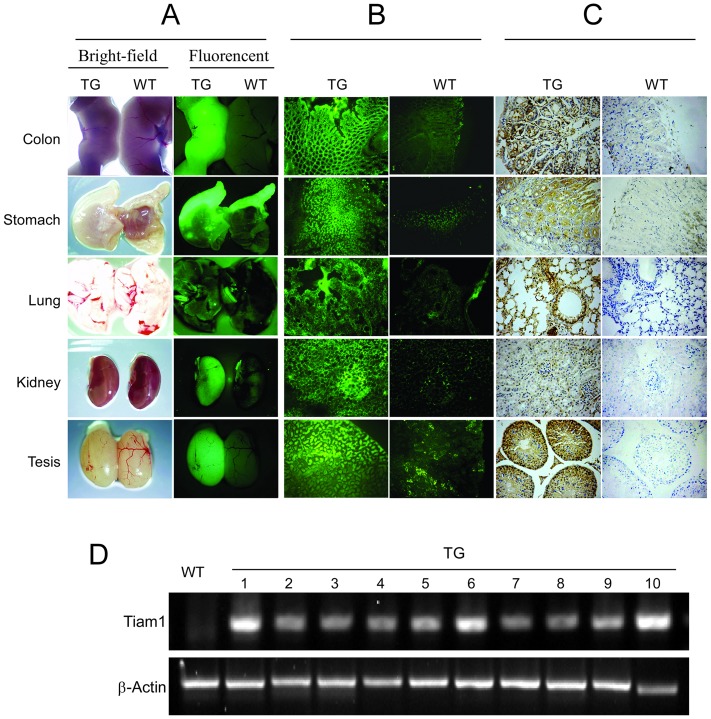
Establishment and identification of Tiam1/EGFP transgenic mice. (A and B) Observation of EGFP and Tiam1 expression in differente organs (A) and corresponding frozen sections (B) from the pCDFl-Tiaml-copGFP transgenic (TG) mice and wild type (WT) mice by a whole-body fluorescence imaging system. (C) Detection of Tiaml protein in the colon of the pCDFl-Tiaml-copGFP transgenic mice and wild type mice through immunohistochemistry staining. (D) Detection of Tiaml mRNA in the colon of 10 pCDFl-Tiaml-copGFP transgenic mice and 1 wild type mice through RT-PCR.

The whole-body fluorescence imaging showed strong GFP signal in the organs of the F1 homozygous Tiam1/EGFP transgenic mice, including colon, stomach, lung, kidney and tesis (Figure 1A). Frozen section of these organs from EGFP transgenic mice also display strong GFP signa (Figure 1B). However, no signal or only weak signal was observed in the wild type mice (Figure 1A and 1B). Immunohistochemical analysis using a rabbit anti-human Tiam1 antibody confirmed that the human Tiam1 protein was strongly expressed in the colon of Tiam1 transgenic mice, while it was only weakly detected in the wild type mice (Figure 1C).

The expression of human Tiam1 mRNA in the colon was analyzed by RT-PCR and could only be detected in Tiam1 transgenic mice with β-Actin used as an expression control (Figure 1D). Treatment with DMH did not induce the expression of endogenous Tiam1 at the mRNA-level, neither in transgenic animals nor in their non-transgenic littermates (data not shown).

### Effect of pCDFl-Tiaml-copGFP on DMH induced Tumorigenesis

**Figure 2:**
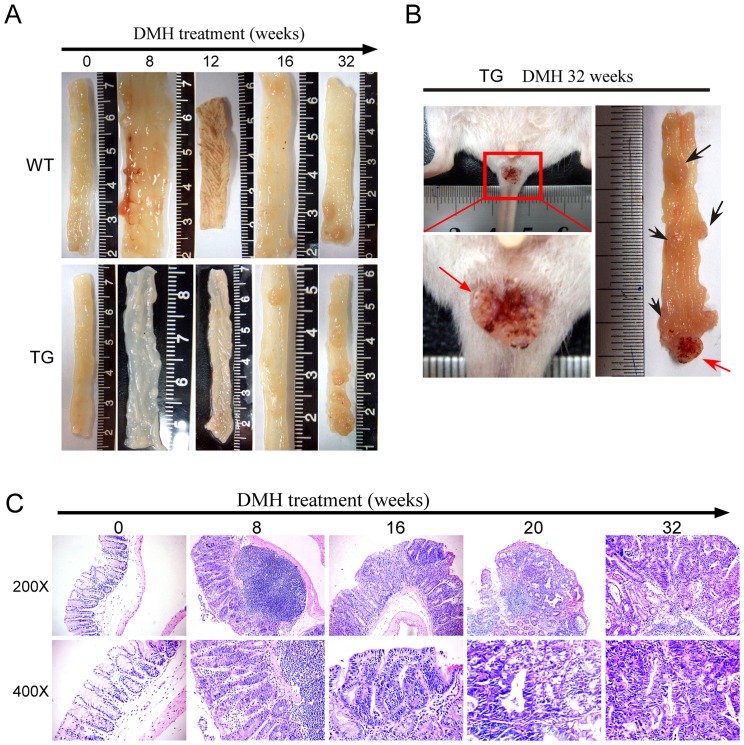
Effects of Tiaml on DMH treatment induced tumor development. (A) Representative Tumors induced under DMH treatment in the colon and rectum of wild-type (WT) (upper panel) and Tiaml transgenic (TG) animal (lower panel) at different time points. (B) Rectal prolapse (arrow) in a Tiaml transgenic (TG) mouse. (C) HE staining of representative tumor lesions in the colon and rectal of wild-type (WT) (upper panel) at different time points.

Eight weeks after DMH-treatment, hyperplasia of lymphoid tissue in the mesentery and intestinal wall in both pCDFl-Tiaml-copGFP (3/5) and non-transgenic littermates (2/5) were observed. Hyperrugosity in the colorectal mucous membrane and thickening of intestinal epithelium occurred in both transgenic (4/5) and wildtype group mice (3/5) at 12 weeks following DMH-treatment. 16 weeks later, colorectal adenoma was observed in both transgenic (2/5) and wildtype group mice (1/5). 20 weeks later, colorectal tumor occurred in both transgenic (1/6) and wildtype group mice (1/6) which was histologically diagnosed as colorectal adenocarcinoma (Table 1). Following DMH-treatment, 21/21 (100%) Tiam1 transgenic mice and 18/21 (85.7%) non-transgenic littermates developed tumors within 32 weeks (Table 1). Figure 2A and 2C showed the representative macro-lesions and histology changes (by H&E staining) occurred on mice colon at corresponding time points. Rectal prolapse was observed in 52.4% (11/21) of Tiam1 transgenic mice (Figure 2B).

**Table 1:** Formation of colorectal adenoma, cancer and distant metastasis in Tiam1 transgenic mice and wild-type mice after DMH treatment.

	Wild type mice (n = 50)						Tiam1 transgenic mice (n = 50)					
Time after DMH treatment (w)	8	12	16	20	24	32	8	12	16	20	24	32
Adenoma	0/5	0/5	1/5	3/6	4/8	3/21	0/5	0/5	2/5	4/6	3/8	0/21
Cancer	0/5	0/5	0/5	1/6	4/8	18/21	0/5	0/5	0/5	1/6	5/8	21/21
Distant metastasis	0/5	0/5	0/5	0/6	0/8	1/21	0/5	0/5	0/5	0/5	2/8	11/21

**Figure 3:**
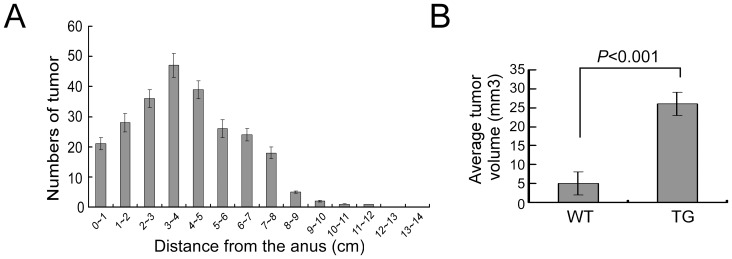
Distribution and tumor volume analyses. (A) Distribution of colorectal tumors in mice after DMH treatment. (B) Comparison of volume of colorectal tumor in transgenic mice and wild-type mice.

The vast majority of the tumors found in the colon were located at 1 to 8 cm from the anus (Figure 3A). In both genotypes, no tumors were found in the small intestine. The tumor incidence and the tumor number per animal were not affected by Tiam1, as were analyzed at 20 weeks, 24 weeks and 32 weeks following DMH treatment (data not shown). However, when average tumor volumes were analyzed on 29 tumors selected randomly from each of the two groups, a significantly increased tumor size was observed in Tiam1 transgenic mice (Figure 3B and Table 2) with a mean tumor volume (23.260±2.119 mm^3^) 4.7-fold higher (P<0.001) than in wild-type mice (4.929±0.580 mm^3^).

**Table 2:** Comparison of tumor volume in Tiam1 transgenic mice and wild-type mice.

Group	n	Volume mean±SEM	t value	P value (2-taile)
TIAM1 transgenic mice	50	23.260±2.119	8.343	<0.001
Wild-type mice	50	4.929±0.580		

### Effect of pCDFl-Tiaml-copGFP on invasive and metastatic ability of DMH induced CRC

**Figure 4:**
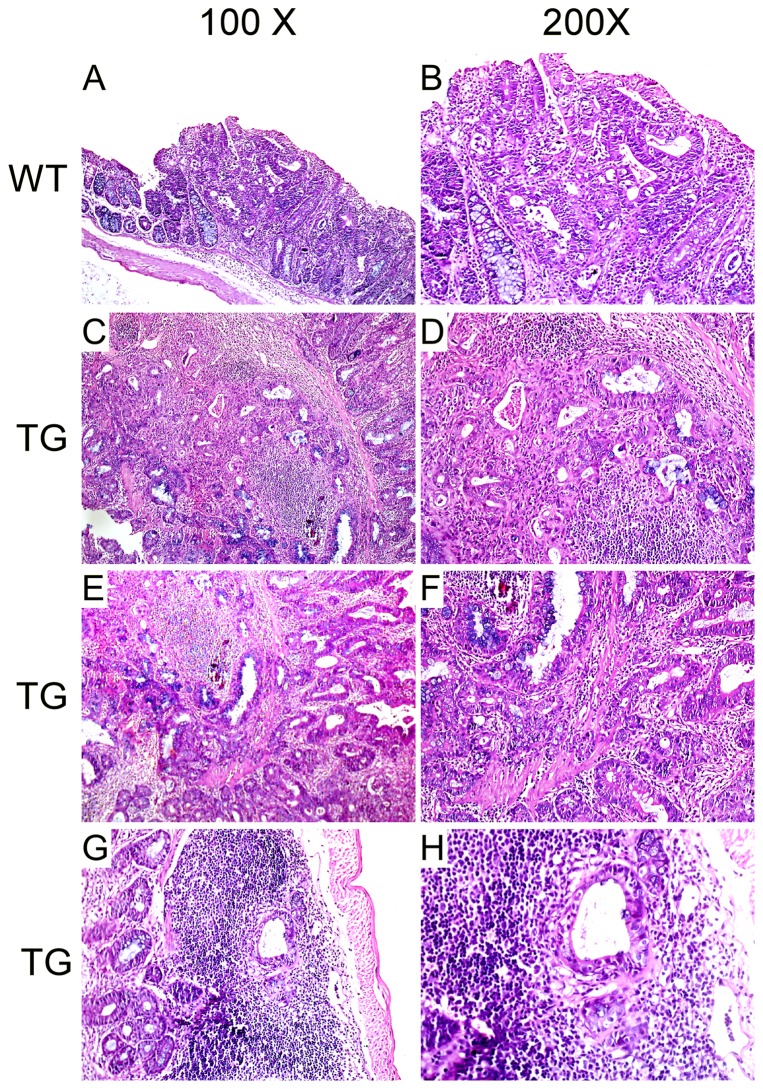
Effect of pCDFl-Tiaml-copGFP on invasion of DMH induced CRC by H&E staining. (A–B) Tumors developed under DMH treatment displayed lesions with only limited depth of neoplastic invasion. The neoplastic glands extended into and through the submucosa, but not abutting the muscle wall. (C–F) Tiaml transgenic mice contained more aggressive tumors and displayed deeper penetration of the neoplastic cells into the muscle layer as well as lymph node within bowel wall (G–H).

To determine the nature of the colorectal masses, we examined the tumors using histological methods. By microscopic analysis, in the non-transgenic mice, most of the tumors developed under DMH treatment displayed lesions with only limited depth of neoplastic invasion. As was shown in Figure 4A–B, the neoplastic colonic epithelium formed a plaque-like mass that was distinct from the adjacent normal mucosa. The neoplastic glands extended into and through the submucosa, but not abutting the muscle wall.

Consistent with the larger volume of tumor developed under DMH treatment, the Tiaml transgenic mice also contained more aggressive tumors and displayed deeper penetration of the neoplastic cells into the muscle layer (Figure 4C–F) as well as lymph node within bowel wall (Figure 4G–H), where they formed distinct glands, as compared with non-transgenic littermates. These aggressive tumors invaded freely through all layers of the bowel forming large mucin-filled glands.

**Figure 5:**
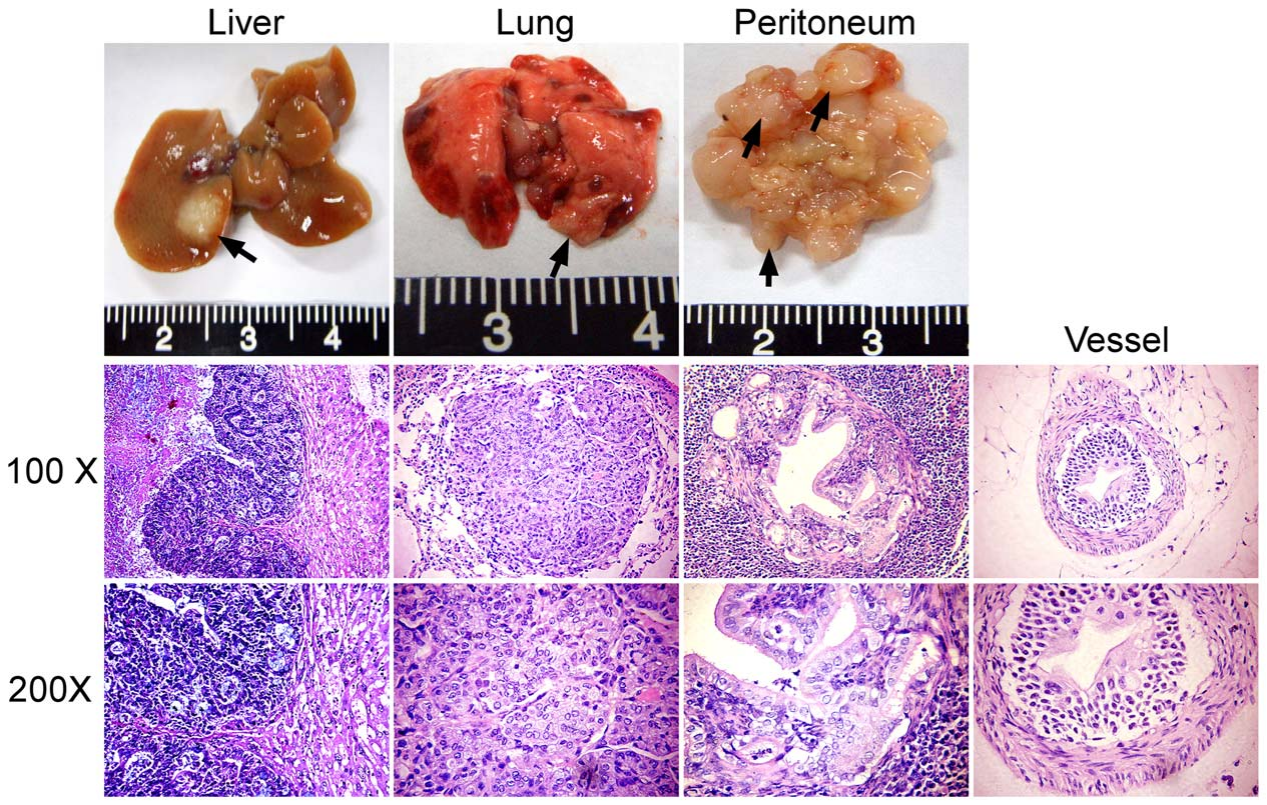
Representative distant metastasis in Tiaml transgenic generated tumors that spread to distant sites, including peritoneum, liver, lung and micro-vessels.

Besides local invasion and lymph node tissue involvement, Tiaml transgenic mice also generated tumors spread to distant sites, including peritoneum, liver and lung (Figure 5). As was summarized in Table 1, 52.4% (11/21) Tiaml transgenic mice generated distant metastasis 32 weeks following DMH treatment. However, no distant metastasis or micro-metastatic lesions were found in all the 21 non-transgenic mice at the same time point (Table 1). Further more, infiltration of tumor cells into small vessels were also observed in Tiaml transgenic mice, while not in non-transgenic mice (Figure 5).

### Activation of the β-Catenin pathway was associated with pCDFl-Tiaml-copGFP transgenic animals

**Figure 6:**
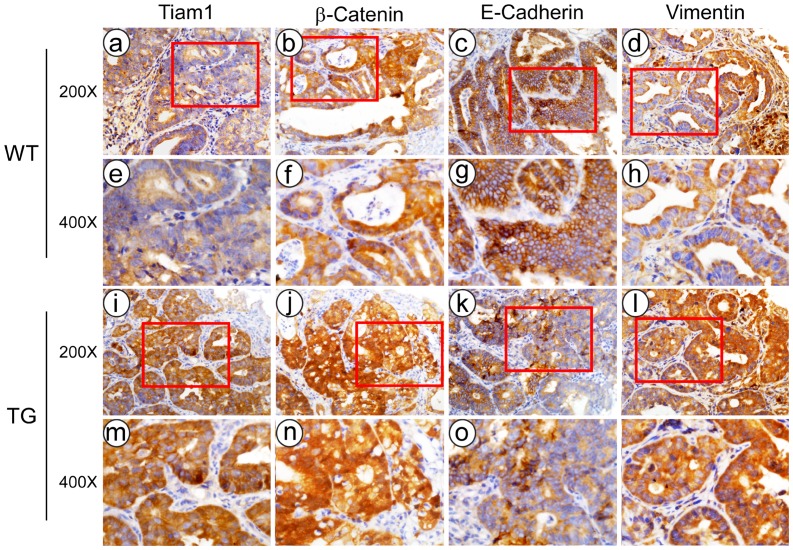
Immunohistochemical staining of Tiam1, β-catenin, E-Cadherin, and Vimentin in cancerous lesions generated by DMH in Tiaml transgenic (TG) mice and wild type (WT) mice.

We next examined the expression of β-Catenin, epithelial marker E-Cadherin, and mesenchymal marker Vimentin in the tumors developed in wild type mice. As was shown in Figure 6, immunohistochemical analysis displayed that tumors formed in Tiaml-transgenic group exhibited higher expression of total and nuclear β-Catenin, and higher weaker staining of E-Cadherin, while stronger staining of Vimentin, as compared with wild type group.

## Discussion

Tiam1 is suggested to play a role in the development and progression of human cancers. On molecular basis, in vitro studies provided insights into the effect of Tiam1 carcinogenesis and tumor invasion. Moreover, in vivo studies showed that Tiam1 involved in cancer development and metastasis in nude mice.

Our previous study has provided evidence that Tiam1 was closely correlated to metastatic potential of colorectal cancer, and its depletion significantly reduced tumor growth and metastasis ability of CRC cells [9]. In order to better understand the role and mechanism of Tiam1 in CRC metastasis in vivo, in this study, we developed pCDFl-Tiaml-copGFP-transgenic mice and induced colorectal tumor formation through treatment of DMH. Through histological analysis, we found that although the tumor incidence and the tumor number per animal were not affected by Tiam1, the average tumor volumes were significantly increased in Tiam1-transgenic group than that in wild-type mice. In addition, we found that Tiam1-transgenic mice formed more aggressive tumors including local invasion and lymph node tissue involvement, as compared with wild type mice. More importantly, Tiaml transgenic mice also generated tumors which spread to distant sites, including peritoneum, liver and lung, as well as infiltration of tumor cells into small vessels. While no distant metastasis or micrometastatic lesions were found in wild type group.

Two opposing functions of Tiam1 on cellular migration, invasion, and adhesion are supported by previous studies in different cellular contexts. On the one hand, it has been reported that presence of Tiam1 tended to be associated with good prognosis in gastric cancer [14]. In Ras-transformed Madin–Darby canine kidney (MDCK) cells and NIH3T3 cells, Tiam1 induces a Mesenchymal-Epithelial transition (MET) and inhibits cell migration through enhancing cadherin-mediated cell–cell adhesion [11], [15], [16]. In human renal cell carcinoma cell lines, Tiam1 inhibits invasion through Rac-induced upregulation of tissue inhibitor of metalloproteinases-1 (TIMP-1) and TIMP-2 [10]. It has been documented that Rac signaling could antagonize Rho activity directly at the GTPase level, and the reciprocal balance between Rac and Rho activity determines cellular morphology, migratory and invasive ability [17]. Therefore, the inhibition effects of Tiam1 on migration and invasion in different contexts might be caused by Rac-mediated suppression of Rho activity.

On the other hand, there are also numerous reports that supported the role of Tiam1 in promotion of malignant transformation, tumor proliferation, invasion, and metastasis [9], [18], [19], [20], [21]. Tiam1 has been implicated in oncogenic transformation of NIH3T3 cells [22]. Additionally, Tiam1 increases invasion and metastasis in several human cancer cell lines including melanoma and breast cancer [18], [23]. Moreover, our previous study also suggests an important role of Tiam1 in migration and metastasis of CRC cells [9]. Our present study provide evidence that the introduction of human Tiam1 gene into the germ-line significantly enhanced the invasion and metastasis of colorectal cancer, while only inconspicuously promotes the development of colorectal tumors which were induced under DMH treatment. Thus, our data mainly support the invasive and metastatic promotion function of Tiam1 in CRC. In yet another study, by comparing tumor biological behaviors in APC mutant Min (multiple intestinal neoplasm) mice expressing or lacking Tiam1, the authors found that Tiam1 depletion caused enhanced invasion of malignant intestinal tumors, although the formation and growth of polyps in vivo were significantly reduced [24]. The evident discrepancy between our present results and the above study might potentially be attributable to the APC mutant background and DMH treatment. Thus, the different effects of Tiam1 on migration and invasion are likely dependent on different contexts such as the cell type, substratum, transformation status, and the activation state of small G proteins in various kinds of cells. To better illustrate Tiam1's role in cancer progression and metastasis, further study is imperative to improve our understanding of Tiam1's upstream regulation.

Wnt/β-Catenin signaling critically regulates development and progression of CRC [25]. Nuclear localization of β-catenin is crucial for canonical Wnt signaling activation. Members of the Rho family of small GTPases, including RhoA, Rac1, and Cdc42, have been shown to participate in promotion activity of Wnt signaling and control planar cell polarity in Drosophila [26]. Rac1 activation induces nuclear translocation of β-Catenin [27]. As a Rac1-specific exchange factor, Tiam1 promotes the interaction of Rac1 and β-Catenin, and is essential for Rac1-mediated activation of the canonical Wnt pathway [28]. In the present study, we demonstrated that introduction of Tiam1 into the germ-line caused a significant increasing of nuclear accumulation of β-Catenin, decreasing of membrane E-Cadherin, and enrichment of Vimentin in the colorectal tumors with more invasive ability. Thus, our present study provides evidence that Tiam1 promotes invasion and metastasis of CRC, and implicates that Wnt/β-Catenin pathway might be involve in the effect of Tiam1 on CRC invasion and metastasis in vivo.

In conclusion, our study has provided in vivo evidence supporting that Tiam1 promotes invasion and metastasis of CRC, most probably through activation of Wntβ-catenin signaling pathway. However, Wnt/β-Catenin signaling pathway could not be the only mechanism through which Tiam1 could promote progression of CRC. Further studies are required to establish the regulatory network of Tiam1 in cancer progression.

## Supporting Information

Figure S1 **Identification of Tiam1/EGFP transgenic mice by PCR.** Transgenic mice and their non-transgenic littermate animals were genotyped by PCR using the following primers for Tiam1: 5′ AAGACGTACTCAGGCCATGTCC 3′ and 5′ GACCCAAATGTCGCAGTCAG 3′. Genomic DNA was prepared from mouse-tail biopsies.
(TIF)
