# Supplementary figures and images for: Tiam1 Transgenic Mice Display Increased Tumor Invasive and Metastatic Potential of Colorectal Cancer after 1,2-Dimethylhydrazine Treatment
Source: PLoS One. 2013 Sep 12;8(9):e73077. doi: 10.1371/journal.pone.0073077 (PMC3771986; doi:10.1371/journal.pone.0073077)

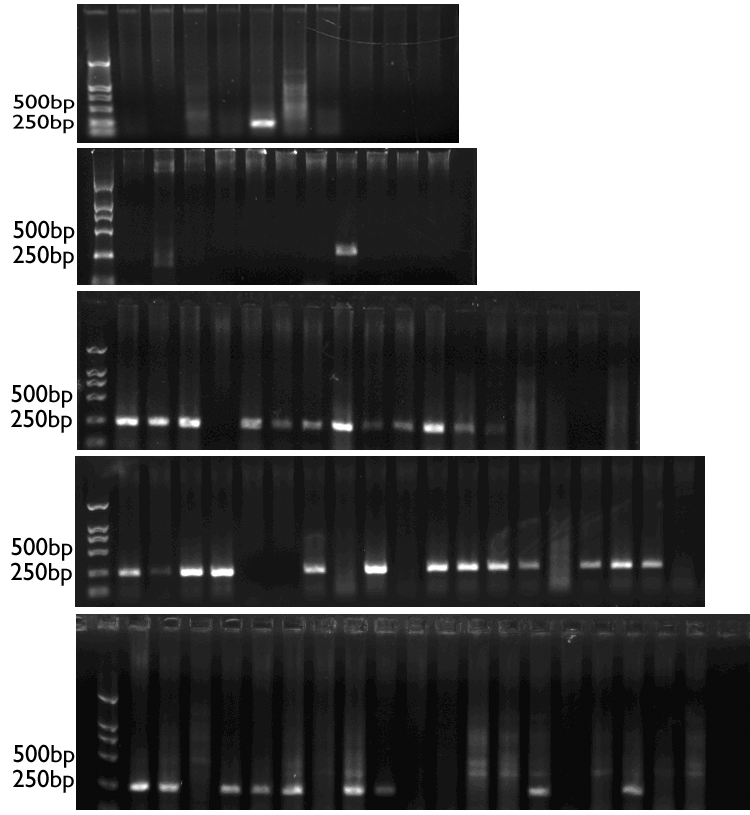

Supplement: Figure S1 — Identification of Tiam1/EGFP transgenic mice by PCR. Transgenic mice and their non-transgenic littermate animals were genotyped by PCR using the following primers for Tiam1: 5′ AAGACGTACTCAGGCCATGTCC 3′ and 5′ GACCCAAATGTCGCAGTCAG 3′. Genomic DNA was prepared from mouse-tail biopsies. (TIF) [file pone.0073077.s001.tif]
